# Create more and more triboelectric charges on polymer surface

**DOI:** 10.1016/j.fmre.2024.01.009

**Published:** 2024-02-07

**Authors:** Xinglin Tao, Xiangyu Chen

**Affiliations:** a CAS Center for Excellence in Nanoscience, Beijing Key Laboratory of Micro-nano Energy and Sensor, Beijing Institute of Nanoenergy and Nanosystems, Chinese Academy of Sciences, Beijing 100083, China; b School of Nanoscience and Engineering, University of Chinese Academy of Sciences, Beijing 100049, China

**Keywords:** Triboelectric nanogenerator, Triboelectric polymer, Charge density, Triboelectricity, Triboelectric property

## Abstract

Electrostatic potential induced by contact/tribo electrification on the polymer surface is often regarded as a hazard to be avoided, since it is usually the cause of electrical sparks and accidental fires. However, this electrification effect can also be an opportunity for energy harvesting and self-powered sensors through triboelectric nanogenerator (TENG) technology. More and more electrostatic charges on the surface of triboelectric polymers are the first challenge for improving the performance of TENGs and achieving various triboelectric applications. This perspective reviews strategies for increasing the charge density and the fundamental principles and conclusions related to the triboelectric properties of polymers.

## 1 Introduction

Electrostatic build-up on the polymer surface, a potential cause of fire and explosion breakdown due to electrical sparks, is often regarded as a hazard to be avoided. However, this phenomenon can be a positive opportunity for various applications such as energy harvesting and self-powered sensors, through triboelectric nanogenerator (TENG) technology [1]. By coupling contact electrification and electrostatic induction, TENG is one of the most promising energy harvesters and can be exploited for various applications such as electricity generators, self-powered sensors, high voltage source and blue energy [2]. In this aspect, perusing high charge density and stable electrostatic on the surface of triboelectric materials is a fundamental and inescapable problem to achieve high performance TENG and various triboelectric application [3]. Polymers are the major materials for fabricating TENGs and the study of triboelectric polymers has attracted tremendous attention owing to their advantages of high charge density, flexibility, cost effectiveness, and light weight [4]. The study of triboelectric polymers is the main study branch of TENGs to fulfil the real application and scale-up manufacturing of diversified TENG devices [5]. Therefore, more and more electrostatic charges on the surface of triboelectric polymers are sought, and ingenious strategies are pursued to increase the charge density of TENG.

On the other hand, the phenomenon of triboelectrification on polymer surfaces still has inconclusive issues, although its widespread use in diverse industries such as electrostatic spraying, electrostatic printing, electrostatic precipitation, and TENG technology [6]. The relationship between the chemical structure and triboelectric performance of polymers is still not clear, which can reveal the reason for the triboelectric capability and further guide the preparation and modification of triboelectric polymers [4]. These issues have been studied for centuries, while they remain poorly understood due to the complexity and lack of research motivation. Since the proposal and development of TENG in 2012 [1], these unresolved issues have gained strong research momentum and new developments. At the same time, polymers are also emerging as electrically functional materials due to their triboelectric capabilities for energy harvesting, sensing, electronic skin, triboelectric catalysis and many other triboelectric applications [7]. Research on triboelectric property of polymers not only benefits the development of high performance TENGs, but can also facilitate the study of many fields related to the polymer materials [7].

This perspective discusses strategies for improving the intrinsic triboelectric properties of materials, suppressing charge decay, and optimizing external factors. It explores the relationship between the chemical and electronic structure of polymers and their triboelectric properties, as well as the underlying physical mechanisms. Further innovations and developments in advanced triboelectric polymers can be inspired here to meet the more complex requirements of TENG and triboelectric applications.

## 2 Strategies to improve intrinsic charge density of triboelectric polymers

**Fig. 1:**
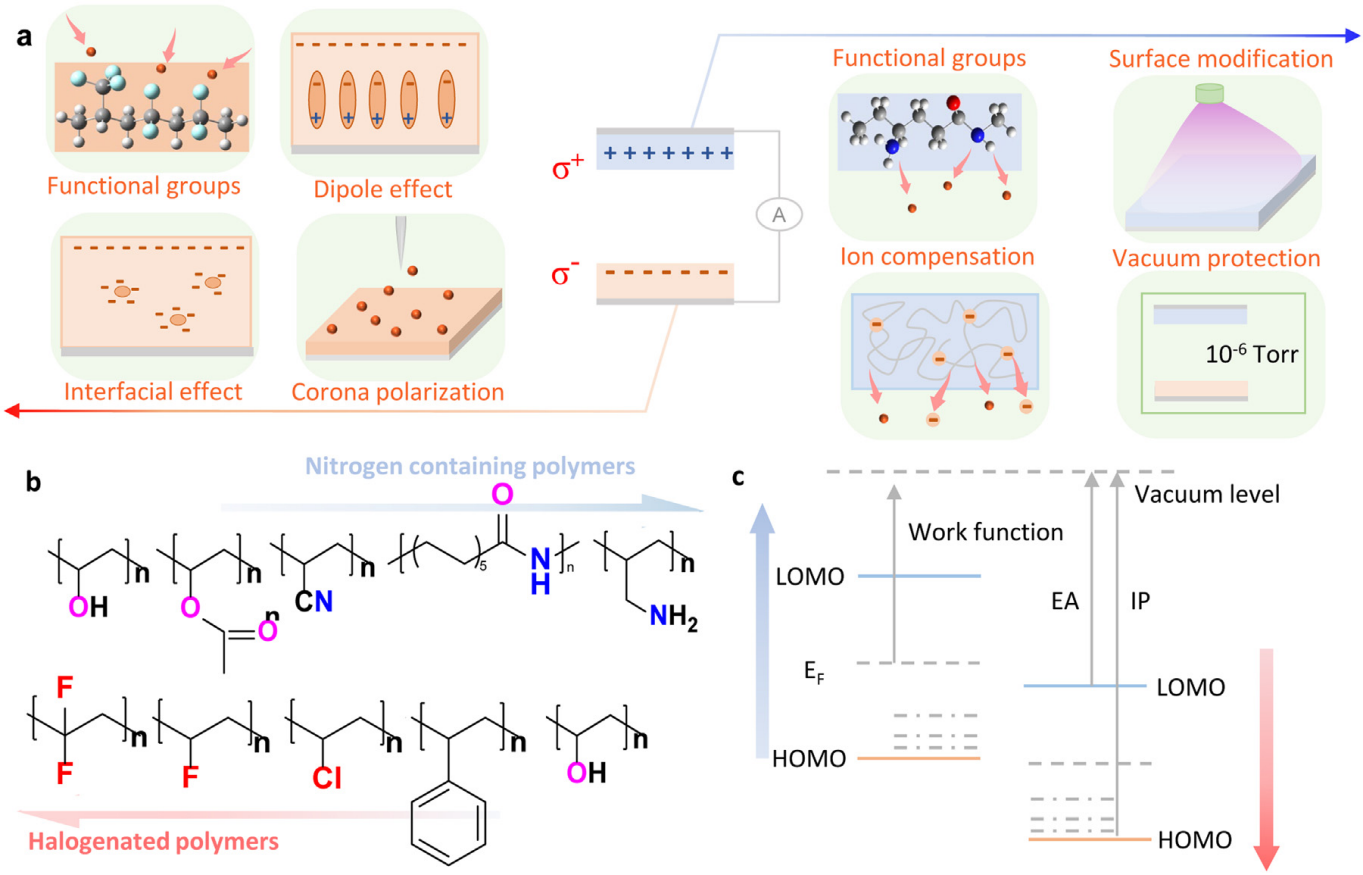
(a) Schematic of material strategies that improve the intrinsic triboelectric property such as functional groups design, dipole effect, interfacial effect, corona polarization, surface modification, ion compensation and vacuum protection. Schematic of the relationship between chemical structure (b), energy band structure (c) and triboelectric property of polymers.

The charge density σ of triboelectric polymers is a critical parameter in determining the output performance of TENG [8]. Previously reported strategies to improve the output performance of TENG can be broadly categorized as: boosting the intrinsic charge density of materials and external optimization. To increase the intrinsic charge density of triboelectric polymers, a series of strategies have been proposed as shown in Fig. 1a. From the chemical structure level, design and synthesis through monomer design, copolymerization and blending in pursuit of superior polarity and high charge density is a good strategy that can fundamentally improve the bulk triboelectric properties of polymers [9,10]. The empirical conclusions can be instructive based on the triboelectric series experimental result that: the nitrogen-containing polymers possess the most positive ranking; the halogenated polymers have excellent triboelectric negative charge properties; and the neutral polyolefins and oxygen-containing polymers develop almost no charge or weak triboelectric capacity. Fig. 1b shows that the types and densities of functional groups with electron-withdrawing/donating ability in the chemical structure contribute to the macroscopic electrification behaviour of the polymer [11]. Surface modification can also alter the functional groups and micro-nanostructures of the polymer surface, but its stability is limited for TENG, which requires continuous friction and contact over long periods of time [12]. To enhance negative polarity, functional groups with strong electron-withdrawing capacity and high electron affinity energy can be introduced. Polymers containing halogen elements typically have a large electron affinity (EA) value, low lowest unoccupied molecular orbital (LUMO) and wide gap (Fig. 1c), which enables strong electron-withdrawing ability and excellent negative triboelectric properties during contact. Polymers with a low ionization potential (IP), high highest occupied molecular orbital (HOMO), and narrow band gap typically exhibit strong electron-donating ability and excellent positive triboelectric properties. The energy band structure is determined by the chemical structure of polymers and is essential for their triboelectric properties. Improving the chemical structure is a fundamental and thorough approach to enhance the triboelectric properties of polymers.

Moreover, enhancing the control of crystallinity and chain orientation also results in an increase in the charge density and polarity of the triboelectric polymer. Controlling annealing, modulating structure, polarizing quenching, and stretching can improve polymer crystallinity. This enhances electron trap and interfacial polarization due to dipole polarization and charge storage at the non-homogeneous crystalline-amorphous interface [13], [14], [15]. These strategies also significantly impact the density of defect states and charge traps in the HOMO-LUMO gap, contributing to the charge density and charge stability. The triboelectric properties of different polymer forms, such as filaments, foams, spun yarns, and aerogels, vary due to changes in aggregate structure, orientation, crystallinity, and surface topology that occur during polymer processing. In addition, adding the appropriate amount of nanofillers can increase deep traps due to dielectric and interfacial effects [16], [17], [18]. Creating interfacial polarization and charge traps can improve the charge density and stability of polymers. These traps can hold more charge and require high activation energy to be excited from them. However, a doping concentration that is too high can lead to charge breakdown and uneven dispersion, resulting in decreased performance. The appropriate doping concentration varies depending on the nature and size of the filler, the substrate, and the means of dispersion. It is important to note that changes in electronic structure are closely related to the study of triboelectric properties of polymers and the preparation of highly polar, high charge density polymers. The relationship between the chemical structure and the HOMO-LUMO structure, electron affinity, ionization potential, and work function has been found to strongly influence the triboelectric properties of materials [19]. It is essential to clarify these mechanisms and principles to understand the physical process of contact electronification and guide the development of high-performance triboelectric polymers.

## 3 Strategy to suppress charge decay

**Fig. 2:**
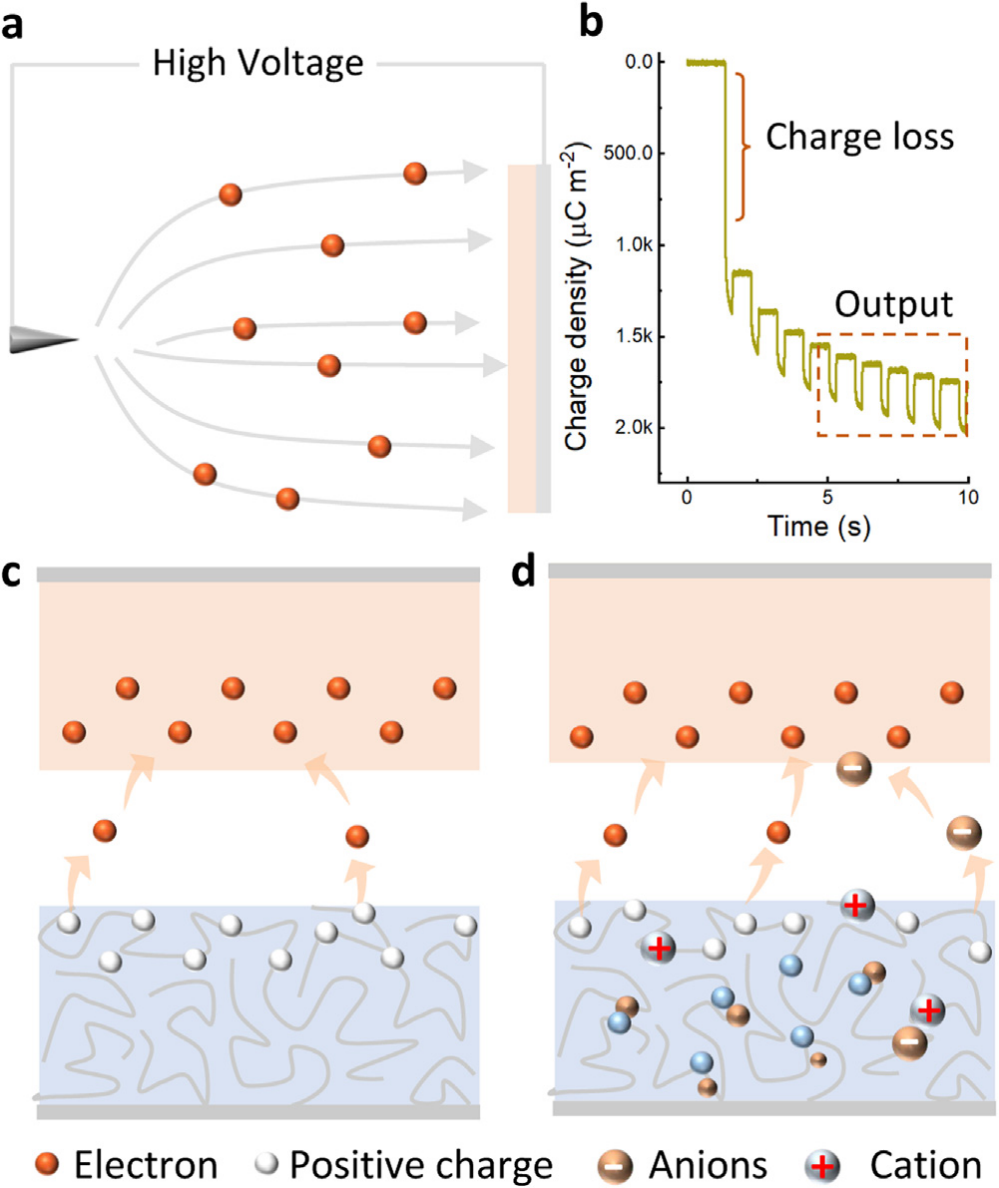
(a) Schematic of corona polarization on electret surface. (b) The output charge density of corona polarized FEP in contact with PA66. Charge transfer mechanisms in contact separation: conventional electron transfer (c) and ionic compensation when ions are involved in ionized surface (d).

Using the methods outlined above, it is possible to achieve an ultra-high charge density on the surface of triboelectric polymers. Particularly, a static charge density up to several mC m^−2^ can be provided on electret surfaces such as fluorinated ethylene propylene (FEP) and polytetrafluoroethylene (PTFE) after corona polarized under a strong electric field (Fig. 2a). However, it is important to note that most of the corona-polarized charges decay rapidly during the contact process, and only a small portion can contribute to the TENG output. For example, the corona-polarized FEP has a 1.5 mC m^−2^ surface charge density, but only 270 µC m^−2^ remains after charge decay during the first few contacts (Fig. 2b). Therefore, the main challenge in improving the effective charge density is to suppress severe charge decay and material wear during continuous contact/friction. Strategies to suppress charge decay and loss can optimize electret benefits to achieve ultra-high charge density in air atmospheres. The focus is on reducing triboelectric charge decay by inhibiting air breakdown, drift, and diffusion of triboelectric charge, and enhancing the charge stability of materials [20]. Some methods such as material modification and chemical structure design can also enhance the charge stability of triboelectric materials [21,22]. However, the effect of related research is not significant. Vacuum protection is currently the most effective strategy for suppressing charge loss, which can reach up to 1250 µC m^−2^ between PVC—Cu pairs under ultra-high vacuum [23]. However, achieving ultra-high vacuum is a major challenge for the flexible and widely used TENG. In the open air, triboelectric materials with ultra-high charge stability and charge retention capability still require considerable effort and dedication. In this regard, triboelectric polymers with ionic participation show significant suppression of charge decay and achieve high charge retention rate, which is one of the most promising strategies to achieve ultra-high charge densities comparable to those in vacuum environments.

As for the fundamental study of contact electrification, it is often debated whether electron transfer or ion transfer is the dominant effect [24,25]. Fig. 2c shows that electron transfer prevails at the solid-solid interface due to the lack of ionic environment. When two materials come into contact, if their electron clouds overlap strongly, the potential barrier between their atoms decreases and electron transfer occurs [26]. Therefore, the charge density and polarity of triboelectric polymers are closely related to their energy band structure as described above. Recent research has found ion transfer and compensation are found on polyelectrolyte or ionized material surfaces, which further enhances charge density and charge stability (see Fig. 2d). The use of radical ion transfer has been reported to achieve ultra-high charge density in ionized-PTFE etched by naphthalene radical anions [27]. Ion-selective transfer on ionized-PTFE surface has also been found to compensate for the charge decay of corona polarization electrets, resulting in high charge retention rate. The electron-ion can synergistically contribute to charge generation and transfer during contact processes, which has been found to result in high charge densities and charge retention capability, especially when in contact with corona-polarized electret materials [28]. This may provide inspiration for obtaining ultra-high surface charge densities in open air as compared to ultra-high vacuum environments. while the mechanisms of charge generation, transfer and decay still need to be studied and explored in depth.

## 4 External optimization strategies to increase output performance

**Fig. 3:**
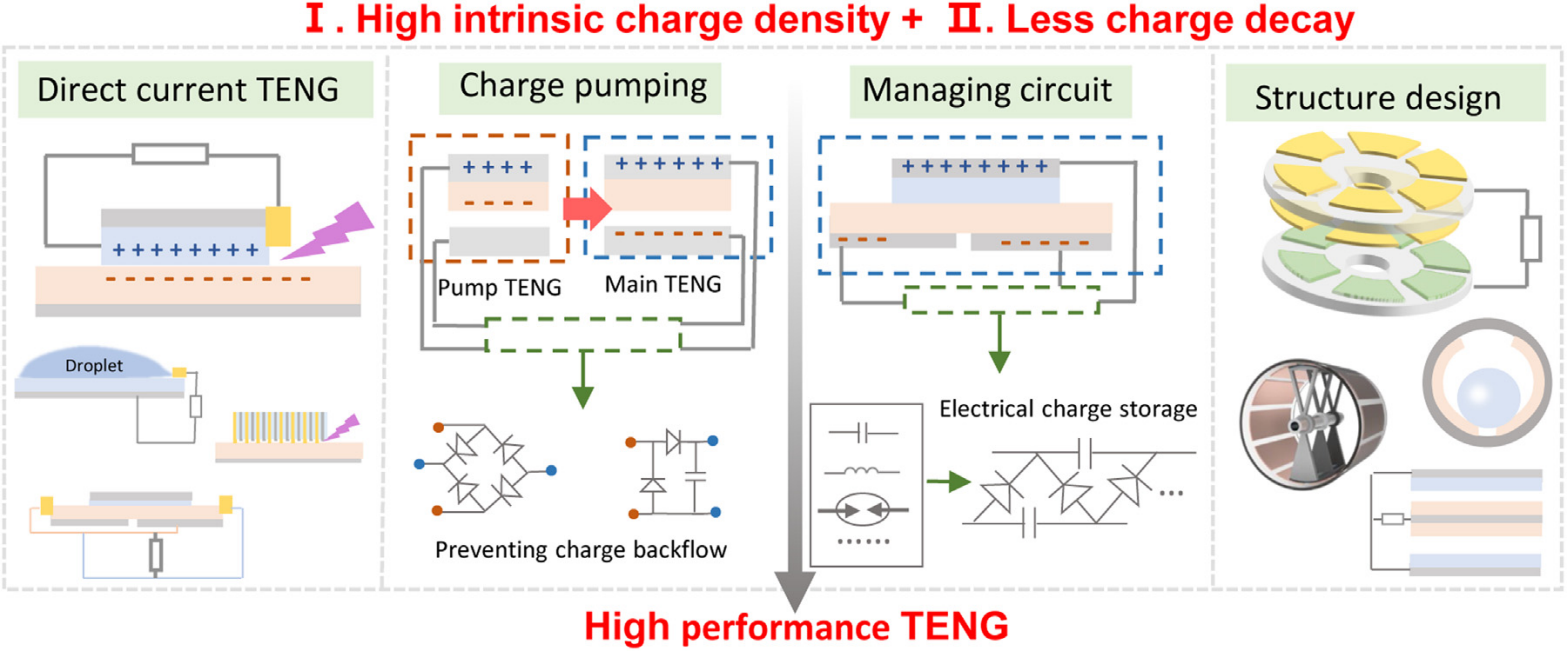
**Schematic of back-end strategies such as direct current TENG, charge excitation, circuit management and structure design**.

It is important to distinguish that the reported charge density is an intrinsic parameter of the material or the TENG output performance by external optimization strategies such as direct current TENG, charge pumping/excitation, management circuit and structure design, which can enhance the TENG output performance and energy conversion efficiency but do not change the material properties (Fig. 3). Previous reports have not been well differentiated and conflated, which is worthy of further study and discussion. The direct current TENG collects some of the electrical energy lost in the air breakdown through the electrodes on the surface of the tribo-layer. Charge excitation can be classified into self-excitation and external excitation, achieved by charge pumping and circuit management respectively. In charge pumping, a pump TENG is used to excite the main TENG and charge reversal is prevented by rectification circuits or other means [29]. In circuits management, this can also be realized by energy storage elements such as inductors and capacitors in conjunction with rectification circuits, also known as self-excitation strategies [30]. Rational structural design of TENG can also better exploit the advantages of triboelectric polymer and good contact conditions, but this is mainly related to the structural figure of merit of the device [8]. These strategies may not affect the intrinsic triboelectric properties of the materials, but are effective in improving the output performance of TENG, which is promising for energy harvesting applications. The development of high-performance triboelectric polymers and low charge decay can take these strategies to the next level of development.

## 5 Conclusion

Charge density is known to be a key metric for polymers to achieve many electrostatic applications such as TENG and sensors. We reviewed the strategies for synthesis and fabrication of polymers with excellent intrinsic triboelectric properties, the strategies to enhance charge retention capability and suppress charge decay, and the external strategies to improve the output performance of TENG devices. Especially, the electron-ion synergy mechanism is expected to break the charge decay limit in corona polarization methods to reach ultra-high charge density in the open air. The research and exploration of triboelectric polymers and the underlying physical mechanisms still require a lot of concrete research progress, which is also of great significance for TENG performance and practical deployment. Further improvements of triboelectric polymers can be achieved by resolving the following challenges:

(1) The combination of economical negative triboelectric polymers that can be polarised and positive polymers with high charge retention has always been the focus of the development of high performance TENG.

(2) The mechanisms and principles of the polymer's triboelectric properties and the mechanisms of charge transfer, movement and loss at the surface require careful in-depth study.

(3) Other functional properties can be integrated into triboelectric polymers to realize the functional and intelligent TENG devices.

We believe that future technological advances towards high performance triboelectric polymers will reveal unconscious physical mechanisms and broad applications in manufacturing and living based on TENG and other electrostatic techniques.

## Declaration of competing interest

The authors declare that they have no conflicts of interest in this work.
